# Health-Seeking Behaviour towards Poverty-Related Disease (PRDs): A Qualitative Study of People Living in Camps and on Campuses in Cameroon

**DOI:** 10.1371/journal.pntd.0005218

**Published:** 2017-01-04

**Authors:** Valerie Makoge, Harro Maat, Lenneke Vaandrager, Maria Koelen

**Affiliations:** 1 Health and Society (HSO) group, Wageningen University, Wageningen, The Netherlands; 2 Institute of Medical Research and Medicinal Plant studies (IMPM), Yaoundé, Cameroon; 3 Knowledge Technology and Innovation (KTI) group, Wageningen University, Wageningen, The Netherlands; Brock University, CANADA

## Abstract

Poverty-Related Diseases (PRDs) emphasize poverty as a ‘breeding-ground’ for a range of diseases. The study presented here starts from the premise that poverty is a general condition that can limit people’s capacity to prevent, mitigate or treat diseases. Using an interpretation of health seeking behaviour (HSB), inspired by the salutogenic approach, we investigated how people deal with PRDs, their ability and strategies put in place to cope. We collected HSB data from two groups of respondents in Cameroon: labourers of the Cameroon Development Corporation (CDC) living in settlements called camps and students of the state universities of Buea and Yaoundé living in settlements we refer to as campuses. By selecting these groups, the study offers a unique view of how different people cope with similar health challenges. We carried out semi-structured interviews with 21 camp dwellers and 21 students in a cross-sectional study. Our findings revealed 1) respondents use multiple resources to cope with PRDs. 2) Respondents’ perceptions of diseases and connection with poverty closely ties to general hygienic conditions of their living environment. 3) Utilisation of health facilities is not strongly dependent on financial resources. 4) Volatile health facilities are a major challenge and reason for people to revert to other health resources. The study brings out the need for organisations (governmental and non-governmental) to strengthen people’s capacities to cope with health situations through better health and housing policies geared at incorporating practices currently used by the people and supporting *pro*-hygienic initiatives.

## Author Summary

People’s living conditions are a crucial factor for health and diseases. In developing countries like Cameroon, poverty is a major condition affecting the way people deal with health issues. We studied people’s a health-seeking behaviour action in two settings: camps, housing labourers of the Cameroon Development Cooperation, and campuses, places where students of the state universities of Buea and Yaoundé reside. We interviewed 21 camp dwellers and 21 students about their health challenges and responses. The results show health challenges not only relate to exposure to diseases but also to the reliability, affordability and functionality of medical services, the hygienic conditions in the living environment and money. For treatment of common diseases, foremost malaria and typhoid, the last resort was going to a clinic or hospital. More prominent responses were cleaning and other hygienic measures, self-medication using available tablets, herbs or other traditional medicine, consultation at small pharmacies or unofficial ‘doctors’. Public health services should anticipate people’s health behaviour to better address the health challenges of people living in poor conditions.

## Introduction

Even though a large number of diseases in low-income countries like Cameroon are preventable and treatable, they still form a big threat to people’s health and well-being. The critical connection between diseases and poverty has caught the attention of national and international health agencies who aim to use this known connection in strategies to reduce the deleterious effects of diseases. The term poverty-related diseases (PRDs) is generally used to indicate poverty as a breeding ground for a range of diseases that can be more easily prevented or treated when living conditions and public services rise to higher developmental levels [1]. The World Health Organisation identifies malaria, HIV and tuberculosis as major PRDs [2]. Cameroon, located in Central Africa, is a nation in which PRDs are a major public health concern. Malaria accounts for most hospital consultations [3]. All people living in Cameroon are at risk of malaria, but the burden is felt more on the poor. The prevalence of HIV in Cameroon is highest in the sub-region of West and Central Africa standing at 5.1% [4]. Also, Cameroon is considered to be medium endemic for tuberculosis [5].

Healthcare insurance policies are almost non-existent in Cameroon. Most healthcare expenditures are paid by family members. Treatment costs therefore imply a major financial burden, especially when health challenges are severe. Some healthcare options in Cameroon include government hospitals and healthcare centres, church-affiliated hospitals and clinics, private doctors, (un)official pharmacies, community health workers and street vendors.

Studies in relation to poverty and health typically argue that people in low-income countries lack financial, material or mental means to prevent disease and do not have access to quality healthcare, and that this results in reduced health and increased disease incidence among the poor [2, 6, 7].

The study presented here starts from the premise that poverty is a general condition that can limit people’s capacity to prevent, mitigate or treat diseases. That notwithstanding, we add a second premise, that people living in conditions of poverty can find ways to deal with health threats. Most research on PRDs works from the first premise only, focusing on limiting factors [8, 9], and little attention is paid to people’s coping mechanisms. In our study, emphasis is put on the latter without ignoring the importance of the first. With our study, we hope to shed more light on what people do to manage their health despite the difficulties they face.

Using the notion of health-seeking behaviour (HSB), we investigated people’s HSB towards PRDs, their ability and strategies put in place to cope. Main approaches within the field of HSB focus either on the process of illness response or on the utilisation of the formal healthcare system [10–12]. In our study, we looked at how our respondents engage in a variety of health-seeking practices to respond to PRDs in their settings i.e. HSB dynamics which we define as the interplay of different factors which influence decisions people take to manage health challenges at subsequent stages in the process of improving their health. We consider HSB more broadly, as actions taken by people to ensure good health in conditions of poverty and how people deal with partly (mal)functioning and partly inaccessible health facilities. To the best of our knowledge, HSB with regard to PRDs have not been reported before, and studies carried out in Cameroon on HSB towards any disease are rare. Insights about HSB, we argue, can reveal unique elements necessary to inform health policies about more integrated forms of health support for people living in poor conditions through building on the agency of local people and supporting community health in more ways than merely offering medical facilities.

Our interpretation of HSB was inspired by the salutogenic approach to health. The principle of salutogenesis, as formulated by Antonovsky [13], is that people’s state of being can be projected on a health continuum, between a state of ease (total health) and dis-ease (total absence of health). He observed that people are constantly confronted with stressors in their daily lives, ranging from psychosocial stressors (e.g. unexpected job loss) to physical and biochemical stressors (e.g. polluted water), and these cause a tension that shifts people’s position on that continuum. Coping successfully with stressors leads to a movement towards the ease (health) end of the continuum, what Antonovsky calls the salutogenic pathway. If stressors are not coped with successfully, stress experienced by people leads to breakdown, either physical or mental—a movement towards the dis-ease end of the continuum or pathogenic pathway [14, 15]. People in Cameroon are confronted with stressors (poverty, disease and so forth) in their daily lives. Being able to deal effectively with these stressors will enable them to move forward and live healthy and fulfilling lives. The salutogenic model helps to refine the HSB model by differentiating between stressors and resources as factors determining people’s strategies and responses to health challenges.

The objective of this study was to investigate HSB towards PRDs in two different groups of people in Cameroon. We collected HSB data from labourers of the Cameroon Development Corporation (CDC) living in settlements called camps and students of the state universities of Buea and Yaoundé living in settlements called campuses. These two dissimilar groups offer a unique view of how different people cope with the same issue. In order to attain our objective, we asked the following research questions:

- What are the health challenges faced by people in camps and on campuses?
- How are health challenges managed by people in camps and on campuses?
- What are the dynamics of HSB in the camps and on the campuses?

## Results

The results section is divided into three parts, presented first for camps and then for campuses where applicable. Firstly, we address health challenges. Secondly, we address HSB in the settings and, thirdly, we address dynamics relating to HSB in the camps and on the campuses.

### Health challenges

Respondents were asked to identify common diseases in their living environments, say which were PRDs and attribute causes for these diseases. The major perceived common diseases were also major perceived PRDs. Malaria was identified as the most commonly perceived disease in both settings. Its presence was attributed to poverty, poor hygiene, poor living conditions, being in the tropics, the high presence of mosquitoes and lack of mosquito nets.

*Malaria is common because it is an illness that is present in Africa… There are those who would always like to get the place dirty. I can say malaria is still present because in some homes the people don’t have nets* …

(Male camp respondent)

Typhoid fever followed malaria in high perceived prevalence and also as a PRD.

*Malaria and typhoid*. *People* [students] *eat outside in poor places because they cannot even manage to eat in good restaurants*…. *Then malaria too*, *because they can’t buy mosquito nets*, *and some of them live in very low-class cités* [student housing] *because of lack of money*.

(Male campus respondent)

Poverty, poor hygiene and bad water were reported as attributed causes for typhoid. Only respondents from campuses named sexually transmitted infections (STIs) as common PRDs.

Other reported health challenges related to financial constraints, quality of treatment received (camps), shortage of drugs at CDC pharmacies and attitude of CDC clinic staff (camps). The nature of poverty in both settings is therefore linked to poor living conditions and finances. Health challenges therefore relate not only to exposure to diseases but also to the reliability, affordability and functionality of medical services.

### Management strategies towards diseases

After identifying the health challenges, respondents reported on how they maintained their health and on how they managed health challenges. These are classified below as health maintenance strategies, informal and formal healthcare strategies.

#### Health maintenance strategies

Hygiene practices came out clearly as efforts employed by respondents to maintain health. In the camps, strategies employed were at both the individual and the community level. Hygiene practices at individual level included re-heating leftover food before consumption to avoid stomach upsets, cleaning clothes, beddings, houses and toilets, and using clean water as much as possible. At community level, hygiene practices reported were clean-up campaigns organised once every month for the general cleaning of camp surroundings.

*We ensure that the compound is neat, we clean up fallen leaves from trees because when there are leaves, mosquitoes hide inside*…

(Female camp respondent)

*We organise special days as clean-up campaigns … the clean-up campaign is held every first Saturday of the month. During this campaign we make sure we keep places clean, especially the taps, because we believe that it is from the taps that we can have cholera-contaminated water*.

(Male camp respondent)

University students live mostly in single rooms. Their reported hygiene practices were mostly at individual level and included keeping themselves, their rooms and their surroundings clean. It also involved using bleach in water carried from wells in cases where there was no pipe-borne water. It was also reported that students in a student building could come together to clean up their surroundings, but this was not as systematic as in the camps. Other strategies reported to maintain health on the campuses were a sporadic medical check-up, using condoms during sexual intercourse, abstinence from sex, taking herbs or prophylactics.

#### Informal healthcare strategies

Informal healthcare strategies were basically deployed outside official healthcare channels and included self-medication, traditional medicine and other informal strategies.

Self-medication emerged as a dominant element under informal healthcare strategies. Self-medication was reported as the first response to any disease threat in the camps, even with available free healthcare services. Self-medication refers to using substances to self-treat self-diagnosed conditions. It usually occurred when illness severity was perceived as low. In the quote below, tablets were used to self-treat a self-diagnosed condition.

*If you have paracetamol you take about three and drink and sit in the house and see what happens…The first thing to do is to try to treat it yourself*.

(Female camp respondent)

Camp respondents reported that, at the first sign of illness, first-aid was administered. This was usually medication found at home (as indicated in the quote above) or bought from informal sources such as street vendors or small pharmacies, or traditional medicine or herbs.

Self-medication was reported more on the campuses than in the camps. At the first sign of illness, campus respondents reported using medication they had at home. The quotes below indicate respondents’ practice of self-medication.

*Here*, *as students*, *there’s a lot of auto medication* [self-medication].

(Male campus respondent)

*For us what happens is that, when someone in the house falls sick, we don’t know what is wrong and so we start first with what we have at home as tablets*.

(Female campus respondent)

Respondents in both settings were able to give the names of drugs they used for self-medication purposes, especially in cases of commonly perceived PRDs like malaria. Coartem (an anti-malarial) was the drug most often named as the treatment taken for malaria. Aspirin, paracetamol and ibuprofen were reported as medicines taken for fevers.

Self-medication was reportedly practiced in both settings using a very similar set of sources: medication that is available at home, buying cheap medication from street vendors and in small quantities (daily doses), buying medication from small pharmacies without a hospital prescription, and consuming bark, roots or leaves of plants (consuming herbs).

Traditional medicine (Table 1) emerged as the second most important element under informal healthcare strategies against PRDs. It was reported in the camps that traditional medicine could come either before hospital care,

*Most times we would do the traditional concoction using mango sticks, pawpaw leaves and when this doesn’t work that’s when we take it to the hospital*.

(Female camp respondent)

or after hospital visit, in which case respondents reported going to the hospital to get a diagnosis before deciding the kind of HSB in which to engage depending on what they were told.

**Table 1:** Reported remedies for treating diseases.

Diseases	Strategy reported	Origin of response
**Malaria**	- Boil together mango sticks, pawpaw leaves and other herbs and drink	Camps/campuses
	- Boil *okongobong* and add a tin of evaporated milk and drink	Camps
**Cholera**	- Salt in a litre of water	Camps
**Typhoid fever**	- Pound 2000 CFA francs (3.5 US$) worth of *okongobong* and add a little water and milk	Camps
	- Mix limes, *okongobong* and water and drink	
**Pains and aches**	- Consume mashed guava leaves	Campus
**Gastritis**	- Consume bitter kola**seed mixed with honey	Campus

*Okongobong is a leafy vegetable common in Cameroonian and Nigerian cuisine

**Bitter kola, or *Garcinia kola*, is a tropical plant with medicinal properties

*As for me, when I discovered that I had typhoid, after the hospital, I came home and started on herb treatment because I know of leaves which can treat the typhoid*.

(Female camp respondent)

There were other reported uses for traditional medicine in the camps. Toilet cleaners reportedly use traditional medicine to ward off the toilet smell from their bodies.

*When I feel the nausea, I go to an old man who will treat me with traditional medicines… He gives me grass to eat and also some to bathe with because, after I clean the toilet, the smell follows me around*.

(Male camp respondent)

Traditional medicine practice was also reported on the campuses. Herbs were mentioned as a first response to disease treatment because of lack of money necessary for hospital consultations.

… *I think for me, most of the guys take herbs as the first step. You see, we are all students and sickness can attack one at every time, and we don’t have money, so the basic step is to spin around and see if you can find the herbs around your house. It works. Something like malaria even, the scientists have done their work but I think the best way to get rid of it is to use herbs*.

(Male campus respondent)

Two other informal healthcare strategies were reported by respondents. One was consultations with quart-doctors for students. Students use the term quart-doctors to refer to doctors in the neighbourhoods (quarter-doctor). Quart-doctors could be a variety of semi-professionals, mostly students still in medical school or young doctors who have graduated from medical school but do not have a place to practice medicine legally.

*In the student areas, there are clandestine doctors and student doctors and these are more affordable than the hospitals*…

(Male campus respondent)

Quart-doctors were reported to run their practice from their rooms or from paid-for extra rooms transformed into offices in student buildings.

The second strategy was consultation at small pharmacies for respondents both in the camps and on the campuses. A small pharmacy is any small shop with some medication in it. It was reported that the person selling medication is called “doctor”, and this vendor could also give prescriptions even though he/she is not a trained medical professional, therefore playing a consulting role similar to the quart-doctors, with the difference that quart-doctors at least have some medical training.

#### Formal healthcare strategies

In the camps, formal healthcare strategies referred mostly to visiting the free CDC clinics (or the CDC hospital in Tiko), which offered free consultations, laboratory examination and treatment. However, factors—for example poor quality of treatment received—hindered full exploitation of this option. Students reported that the last-resort option for seeking health was to go to the hospital. Hospitals around the universities were both public and private and required payment for services to be made fully and in advance, thereby reducing their appeal for students who were already under financial constraints.

### Dynamics of health-seeking behaviour

**Fig 1:**
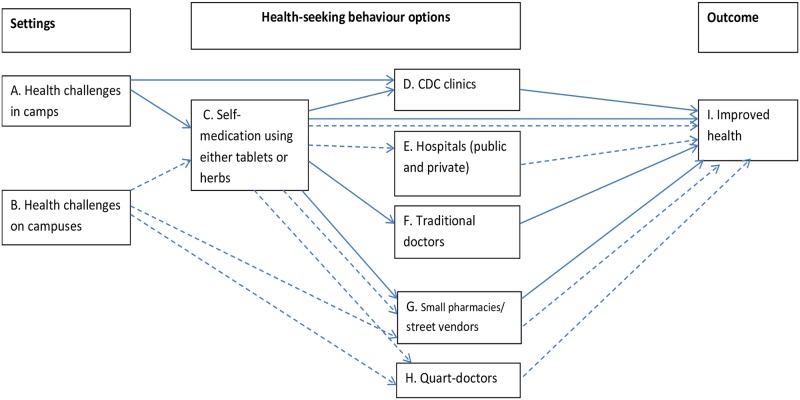
Schematic model of dynamics of HSB in camps and on campuses.

A major finding in our study was that HSB appeared very dynamic (see Fig 1). Factors behind the dynamics were local perceptions, perceived disease severity and financial constraints.

#### Local perceptions

Local perceptions of diseases influenced HSB. There were some diseases that respondents believed could not be treated with tablets but could be treated with herbs. Examples of such diseases were malaria and typhoid fever, both of which were commonly perceived PRDs in their living environments. The following quotes illustrate this perception.

*I know that there’s no tablet which treats malaria. All the tablets do is reduce discomfort. It is only traditional medicines that treat malaria. They can give you tablets to reduce discomfort for a month or so, but after that any other attack is more severe. So for me, I think that, if one has a malaria attack, it is best to go directly to the traditional way. You will be treated when you go traditional, and malaria will not be a problem for you anymore*.

(Male camp respondent)

*We take herbs especially when it is a typhoid case. People say that the hospital can’t treat that, they can only calm it down and so, in such cases, herbs are the way to go*.

(Female campus respondent)

#### Perceived severity of disease

The perceived severity of the disease, whether high or low, reportedly influenced whether HSB was formal or informal.

*Except when it is very serious that I can rush to the hospital* [CDC clinic].

(Female camp respondent)

*I go to the hospital depending on the severity of the illness or procedure*.

(Male campus respondent)

The preferred alternative in both settings when illness was not perceived as severe enough to go to the clinic or the hospital was home treatment (self-medication) or buying drugs from street vendors and small pharmacies. This happened even in the camps despite freely available healthcare, and even when these were out-of-pocket payment alternatives.

…*There are other good antimalarial drugs and not expensive… I bought Artequin for 500 CFA francs and it was effective. When somebody is sick, I always go immediately to the CDC clinic. I go there so that they can detect what is wrong. If malaria is detected, I will go and buy drugs from somewhere else*.

(Male camp respondent)

#### Financial considerations

Financial constraints also influenced the type of HSB that people adopted. Formal HSB was reported more in the camps than on the campuses. Camp respondents reported that, as healthcare services were free for them, they were more likely to consider going to the CDC clinics when ill.

…*when you go to other hospitals, whatever they have to do, they ask for money: consultation, lab etc., but since the CDC clinic is where we work and as it is free for us, we quickly go there*.

(Female camp respondent)

However, camp respondents engaged in a careful self-diagnosis and weighing of options before turning to the clinic. Awareness about the poor quality of the treatment received at the clinics was set against local perceptions or the perceived severity of the disease before the decision to go to the clinic was taken.

*The medicine that is given* [at CDC clinics] *for malaria breaks one down*, *it makes one restless*, *and blocks ones ears*.

(Female camp respondent)

Students reported that they would rarely use formal healthcare (hospitals) because of financial constraints, but also when disease severity was perceived as low. For these reasons, they engaged in self-medication and other health-seeking strategies such as recourse to quart-doctors who were their informal healthcare providers and an alternative to both formal hospital care and street-vendor healthcare. Students reported using quart-doctors because of proximity, affordability and also payment flexibility (accepting collateral such as national identity cards instead of immediate cash payment).

…*students have no money; they can’t borrow medicine from there* [hospitals]. *In that case*, *the students are forced to go to quart-doctors*.

(Male campus respondent)

Although cheaper than going to the hospital, participants reported a need for caution when consulting with quart-doctors.

*Quart-doctors are those people who open clandestine medicine shops who have not been authorised to practice*. *Quart-doctors are common in Molyko* [place where University of Buea is located]. *Quart-doctors do carry out abortions even*. *You know the rate of unwanted pregnancies in Molyko is very high*. *Quart-doctors are very cheap because they are out to make money*…

(Male campus respondent)

Consulting at small pharmacies was another option followed because students could not afford to go to the hospital. This usually came after the failure of self-medication using tablets at home or herbs. Small pharmacies were reportedly appealing because they were cheaper and flexible in selling daily doses when requested.

*When I was sick with malaria, I went to the pharmacy to buy drugs since we are in school and cannot afford to go to the hospital and do consultation so we go to the pharmacy and just consult there and they prescribe to us what they can*.

(Female campus respondent)

Finally, even though students said that they did not rush to the hospital, they reported situations that would make them do so, for example when the illness severity was perceived as high; this usually occurred when they had not succeeded in treating themselves in the ways described above. Strategies reported for paying for healthcare included loans from friends and support from parents and relatives.

*If you feel too bad, you go the hospital, maybe you start from pharmacies before eventually going to the hospital when it is serious. People will first of all start with self-treatment and, if it doesn’t work, then they will go to the hospital*.

(Male campus respondent)

## Discussion

The aim of our study was to investigate HSB towards PRDs in two different groups of people in Cameroon. In both groups, there was a clear interaction between challenges in general living conditions, finances and health. Our findings show that malaria and typhoid fever are perceived as major PRDs, with a clearly expressed link to poor hygiene as the root cause. In both settings, people’s response to health threats, and practices to prevent and to quickly overcome disease, typically demonstrate an eclectic use of available options and services. Official healthcare, in the form either of free CDC clinics as in the camps or of paid-for public hospitals for students, is only one of the available options. There is also a variety of self-medication practices and services and medication from unofficial sources, resulting in a hybrid platform for HSB. The case of the CDC clinics shows that free healthcare services do not stop self-medication practices and the use of alternative healthcare. Below, we discuss key findings of our study.

### PRDs

As in other developing countries, malaria was perceived as a common disease in both settings [16–18]. Its presence was attributed to poverty, poor hygiene and tropical climatic conditions conducive to mosquitoes. Typhoid fever was also perceived as a major PRD in both settings. This is not in line with the WHO [2] classification of major PRDs, which does not include typhoid fever. Our results suggest that more attention should be paid to diseases linked to hygiene in research into PRDs and policies formulated to fight PRDs. Situation-specific hygiene conditions are a potential source of diseases, but our findings show that people understand the importance of good hygiene and can mobilise to improve their immediate living environment. However, the perceived persistence of diseases suggests that the efforts made by people may not be sufficient to significantly reduce the PRD burden. Health policies must be geared towards actions in support of people’s initiatives to improve their living conditions.

Malaria and other PRDs pose burdens for the health and well-being of people in the studied settings, and conditions of poverty only serve to aggravate such burdens. Our study has created a better understanding of how two dissimilar groups of people, in terms of education and occupation, manage very similar conditions in which lack of financial resources and general poverty affect health. Our findings show that people find versatile and creative ways to combine available resources to improve their health and well-being, i.e. a move towards the ease end of the salutogenic ease—dis(ease) continuum. The purpose of a HSB study is usually to find out how people interact with health systems—the hindrances to, and facilitators of, engaging with formal health systems [12]. In our study, we have gone further and additionally looked at how people manage means at their disposal. We have looked at both formal and informal HSBs as well as the dynamics at play as people seek health.

Our study showed that HSB could be influenced by many variants, such as the attitude of staff (camps), quality of treatment, perceived severity of disease, respondents’ (lack of) money and also respondents’ local perceptions about diseases. In the following sections, we discuss respondents’ management of diseases as well as the dynamics underlying HSB.

### Management strategies

The people in the camps and on the campuses demonstrated unique ways of dealing with PRDs and other health issues, and it was interesting to find similarities among two such different groups.

In both settings, self-medication was indicated as a first response to illness, and this was not a function of education (since both educated and uneducated respondents were involved in the practice) but rather of poverty, perceived disease severity and the volatility of health services. Volatile health facilities refer to facilities which are not consistently functional, accessible or affordable. This refers especially to camp situations in which people are ‘entitled’ to free treatment and medication but these are often unavailable and patients are asked to come back several times requiring them to pay transport for that for which they do not have the money and so they stop going.

Self-medication is a reported practice in other countries as well [6, 7, 19, 20]. What our study shows is that people are knowledgeable about the treatment needed for their illnesses. The capacity for self-diagnosis and subsequent action to find the required treatment was not limited to official clinics or pharmacies but rather reflected a much wider array of treatment options. When asked about this, people were able to report various recipes as treatment for malaria, typhoid and so forth, and (brand) names of pills for these diseases. Further research could investigate the effectiveness of the different recipes reported as treatments. Even though self-medication was practiced in both settings, it was more common in the student milieu. This is probably because students face more financial constraints and weigh their options for spending money on medication against various other expenses. The situation in the CDC camps, however, makes clear that free healthcare services do not eliminate self-medication (first-aid) and the use of alternative sources by camp respondents. Factors such as poor quality of drugs received at the clinics were reported as main reasons for self-medication. The side-effects associated with these drugs did not permit respondents to complete the treatment and also resulted in a preference for drugs from other sources or the use of herbs. Respondents also reported frequent drug shortages at the CDC pharmacy, requiring either several trips to the clinic or, the preferred option, a shift to self-medication. Shortage of drugs at health centres has also been reported in other countries like Tanzania as a reason for self-medication [21, 22]. Another reason for choosing self-medication over free clinics was the reported poor attitude of the medical staff. The way people are received in healthcare services and treatment quality are therefore important issues to be considered by CDC and other health providers in order for the services they provide to be effectively used [23]. From the above, we can see that self-medication was considered for several reasons, mainly to save time and money, to have a choice about the kind of medication to take and to have control over a health situation. However, using self-medication as a first response to disease could lead also to delay in seeking appropriate treatment if it fails [12].

Traditional medicine was another way in which respondents primarily managed diseases. This was reported as seeking a traditional healer or as that part of self-medication in which the respondents used plants (herbs). This type of HSB was common in both settings, as respondents unanimously reported their use of herbs to prevent and treat diseases. Herbs were reportedly considered an enticing alternative because they were accessible, cheaper, natural and devoid of chemicals found in tablets that cause severe side-effects (as reported in the camps). Herbs were used to treat malaria, typhoid and aches. This finding adds to previously reported studies on the use of herbs for treatment of conditions such as gynaecological complaints [24] and liver diseases [25].

### HSB dynamics

The dynamics of HSB were found to be based mainly on three crucial aspects. These were: local perceptions of disease, perceived severity of disease and financial considerations.

#### Local perceptions of disease

It was important to understand in our study perceptions people had towards diseases and treatment options, as it was clear from their reports that these perceptions influenced their HSB. Other studies have shown that local beliefs can influence whether respondents accept medical treatments for a disease or not [26–28]. In fact, local beliefs have been reported to influence even the perceived causes and recognition of symptoms for diseases [27]. In our study, malaria and typhoid were diseases that respondents believed could not be treated using tablets. The response to malaria and typhoid was therefore guided by these perceptions. HSB then leaned towards what was expected to bring cure, and in our study this was herbal remedies. It was only when this route did not work and the disease was perceived to be more severe than thought that other options were considered.

#### Perceived severity of disease

It was also important to understand at which point disease was considered as severe in our study, because perceived severity was a factor influencing HSB [29]. The decision to seek healthcare took into consideration whether disease severity was perceived as low or high [30]. Once the severity of the disease was established, the decision on a formal or an informal next step was made. The respondents indicated that illness was considered severe when the first measure—self-medication—to treat it failed to bring relief. As in other countries, self-medication was common when illness was not perceived as severe [30–31]. This was done using drugs available at home, home concoctions with herbs or drugs bought at small pharmacies. This practice was seen both in the camps and on the campuses. When severity was perceived as high, multiple factors influenced the path of action pursued, including questions of where to go next, how much that option would cost and whether there was cost/payment flexibility.

#### Financial considerations

Respondents tended to avoid hospitals, which they described as being expensive and strict in payment options offered. Collateral was not accepted and instead upfront payment was required. Hospitals were therefore not visited until conditions worsened and became unbearable. Finance for healthcare was crucial to determine whether or not to seek treatment and which kind of treatment should be sought. The high cost associated with visiting hospitals was indicated as justification for not going, except in cases of high perceived severity [32]. The healthcare provider’s flexibility in relation to payment was an important factor in HSB, especially on the campuses. Health providers who were not strict about cost of treatment and payment options were more appealing options than those who demanded prompt and full payment [30]. Payment flexibility and accessibility made quart-doctors very appealing for students. Flexibility in selling small doses at cheaper rates made small pharmacies and street vendors enticing for people in camps and on campuses.

### Methodological reflection

In this study we did not focus on gender-based management strategies but rather settings-based (camps and campuses) management strategies to health challenges. We therefore have not reported information (if any) of existing differences in the way males and females respond to diseases. An analysis of gender-based responses to health issues within the settings would be interesting for further research. That notwithstanding, we have provided information on diversity in respondents in terms of age, education and occupation and how this relates to their response to health challenges in the settings.

By purposefully selecting respondents with different jobs and students of different study programs, we acknowledge an element of researcher bias. However, this is common with qualitative studies which seek respondents who have information or experience with the phenomenon under study. Also, since we were not getting any new information with newly recruited respondents, we believe that we have covered the experiences of most people living in the camps or on campuses.

We recognise that one of the limitations of a qualitative study is the reduced possibility to generalise the findings across the wider population. That notwithstanding, qualitative studies such as ours offer an opportunity to go in-depth in the case studied and bring out rich dynamics which will hardly be captured by quantitative studies. Our selection of two research sites, camps and campuses, was not meant as a comparative study but as a way to gain deeper insight into the variety of ways poverty is a co-determinant for health and disease. By doing so our study provides rich information on the types of health challenges the people in camps and on campuses have and also how they manage these challenges. We also acknowledge that this information cannot be generalised across other groups in Cameroon which may have their own health seeking strategies.

### Conclusion

The aim of this study was to investigate HSB towards PRDs in two different groups of people. Our findings have revealed remarkable insights into the reasons behind HSB and strategies used. We started from two premises: 1) poverty is a general condition limiting a person’s ability to prevent, mitigate or treat diseases; 2) people living in conditions of poverty can find ways to manage health threats and improve their conditions. Our study showed that people’s own perceptions of diseases and the connection with poverty were closely tied to the general hygiene in their living environment. For example, the presence of malaria and typhoid were attributed to poor hygiene. Moreover, access to health facilities was not a clear-cut situation dependent on financial resources. Our findings showed that volatile health facilities are a major challenge and a reason for people to have recourse to other resources. Regarding people in the camps, despite free healthcare services offered by CDC, respondents reported using self-medication as a response to disease, showing that the interaction between poverty and HSB is much broader than financial means. With regard to the students’ situation, a specific grey zone of healthcare was visible in the form of quart-doctors, in most cases people with some medical training at academic level but without official positions as medical doctors. The results from both settings show a substantial level of perceived knowledgeability and control over personal health conditions. The versatile response, using a variety of resources in a complementary way, is the main mechanism associated with HSB in the camps and on the campuses. The results of this study are intended to promote the need for organisations (governmental and non-governmental) to strengthen people’s capacities to cope with their health situations. This can be done on the one hand through better health policies geared at incorporating and improving self-medication and traditional practices currently used. On the other hand, through improved housing policies geared at supporting initiatives people have in place such as the clean-up campaigns reported in this study. A focus on capacity building at a societal level by CDC and the Cameroon government is essential to fill the gaps left by systemic weaknesses of the extant healthcare system revealed in this study.

## Methods

### Ethics statement

This study was approved by the Wageningen University review board and the Human Resources and Health departments of CDC. The aim and the procedure of this study were explained to all respondents who met the inclusion criteria. Respondents were informed of their right to leave at any stage without explanation. Respondents were assured of anonymity, and each respondent signed an informed consent form before participating in the study.

### Characteristics of the study settings and population

Cameroon is located in Central Africa. The country is divided into ten regions with about 22 million inhabitants. This study was conducted in the camps and on the campuses providing housing to wage labourers of CDC and to students of the state universities of Buea and Yaoundé, respectively. The settings were selected because 1) they are both host to people originally from different parts of Cameroon settled in the settings for work or studies, respectively, and 2) differences in the participants’ socio-demographic characteristics offered an opportunity to see different people’s responses to PRDs.

The first setting (camps) was made up of three camps (Limbe camp, camp 7 and Sonne camp) all belonging to CDC, a parastatal agro-industrial company with plantations in the southwest region of Cameroon. CDC employs about sixteen thousand people, the majority of them being low-paid, low-educational-level wage labourers who live in camps provided by CDC [33]. Camps vary in size, housing from scores (e.g. camp 7 and Sonne camps) to hundreds (Limbe camp) of families and are similar in terms of housing, activities and living conditions. CDC offers its labourers and their family members free healthcare services in CDC clinics (or the only CDC hospital, in Tiko, Southwest Cameroon). These free services include consultations, laboratory examination and medication when available.

The second setting (campuses) was made up of two universities: University of Buea (UB) and University of Yaoundé (UNIYAO) found in the Southwest and Centre regions, respectively. UB has a population of over twelve thousand students [34], and UNIYAO has over thirty-three thousand students (2007 estimate) [35]. Most students live in neighbourhoods in the vicinity of the campuses. Students can rent rooms either on-campus or off-campus, but most rent off-campus because, on-campus, available rooms are few for the high demand. Student neighbourhoods are similar around both universities in terms of housing, activities and living conditions. For healthcare, unlike in the camps where it is provided free by CDC, students have to pay for the services themselves off-campus.

### Study design and respondents’ characteristics

This study was part of a larger research project, focusing on a wider set of health-related factors and responses from people to health challenges in the two research sites. The overall objective of the study is to understand how conditions of poverty impacted the health of people and how they managed these challenges. The study reported here was cross-sectional in design and took place in 2013. Semi-structured interviews were conducted using an interview guide with 21 camp dwellers and 21 students. In the camps, the respondents were fourteen males and seven females. Fourteen of these had basic primary education, three had no formal education, three had secondary education, and one had high school education. Twelve respondents from the camps were married, six were single and three either separated or widowed. On the campuses, there were 16 females and five males. All campus respondents were single.

In order to be eligible for the study, a person had to be living in the camps as a worker, or a dependant of a worker (camp respondents), or a student of UB or UNIYAO (campus respondents). Respondents from CDC camps were employed as tappers, camp and compound cleaners, security guards, weeders or dependent household members. Students interviewed were part of programmes such as educational psychology, journalism, diplomacy, English, life sciences and so forth.

### Procedure

To reach different job categories and study programmes, respondents from the camps were selected after consultations with camp supervisors, and students were selected after consultations with student leaders. Selection of respondents purposefully weighed towards camp dwellers who were CDC workers and university students living in the Molyko and Ngoa-Ekelle neighbourhoods because respondents in these areas had experience with the way CDC or health services around the universities worked respectively. Camp supervisors and student leaders were entry points into the settings. These were members of the student community, in case of campuses, and CDC employees in case of the camps, known to and knowing the people.

Recruitment sites were the houses of camp dwellers and student buildings. Respondents were introduced to the researcher and the research and consent was sought for participation. Respondents anonymity was assured during this process. All respondents approached agreed to be interviewed. Data collection stopped when no new information was obtained with newly recruited respondents [36].

### Interview guide

An interview guide (S1 Text. INTERVIEW GUIDE) was designed after preliminary visits to the camps and the campuses, conversations with health overseers of the camps and student leaders, and observations of activities in the settings. Observations were made of meetings that usually take place each morning before workers go to the field, meetings of CDC workers, meetings with health overseers and camp supervisors, general daily activities in the camps and on the campuses. The interview guide was designed on the basis of the observations, HSB literature, the health continuum of the salutogenic model [12, 37–40] and sought to identify PRDs and other health challenges and how people coped with them. The first part of the guide had background or entry questions ascertaining the demographic characteristics of the respondents. What followed were questions to identify health challenges, enabling respondents to reflect on how they managed these challenges. Most of the interviews were conducted in English. Pidgin-English was used in interviews when preferred in the camps. Interviews were conducted in the camps by the first author, assisted by a CDC head office junior worker. Interviews on the campuses were conducted by the first author assisted by a trained student assistant.

### Data analysis

The interviews were audio-taped, and detailed field notes were also taken. Interviews in English were transcribed verbatim style. Pidgin-English interviews were translated into English as they were being transcribed. Data were analysed using the ATLAS.ti 7.5 software (Scientific Software Development). Thematic analysis was carried out following Braun and Clarke's [41] protocol. The analysis process started with reading the transcripts several times and then generating codes. The transcripts were printed, read over several times for familiarisation with the data and then coded twice; first on raw transcripts and then in ATLAS.ti. Coding was guided by the salutogenic health model [13] and HSB literature [12, 39, 40]. The interest for the paper presented here was HSB. Braun and Clarke’s protocol [41] permit the possibility of flexibility in identifying themes in several ways. We identified themes based on our research questions. This implies we did a top-down coding. For the interest of our study, it was important to first of all identify the health challenges faced by respondents from the data. This made up one theme and the codes were common diseases and PRDs. We then identified general themes relating to HSB reported in literature from the data. These themes still followed from our research questions i.e. how health challenges were managed by people on camps and campuses. To answer these, we coded following practices such as self-medication, traditional medicine, using small pharmacies etc. From the data, we could see emerging patterns in health seeking behaviour and these were abstracted as dynamics of HSB in the camps and campuses.

Thematic analysis revealed that health challenges faced by respondents related to the presence of diseases such as PRDs, living conditions and healthcare services (functionality, accessibility and affordability). Management strategies revealed are categorised as informal and formal healthcare strategies, as shown on Table 2, alongside key elements associated with that theme. Further analysis of health challenges and management strategies revealed detailed patterns of HSB, highlighted and illustrated with quotations to explain why people do what they do health-seeking-wise, i.e. HSB dynamics. These related to local perceptions (effectiveness of medication to treat disease), perceived severity of disease (low or high) and financial considerations (flexibility of payment options).

**Table 2:** Health management strategies and identified elements emerging from the interviews.

Health challenge management strategies	Identified elements
Informal healthcare strategies	a) Self-medication b) Traditional medicine (herbs or traditional doctor) c) Buying from small pharmacies/road vendors d) Consultations at quart-doctors* or small pharmacies
Formal healthcare strategies	a) Going to CDC clinics b) Going to hospitals

* Students still at medical school or medical graduates not yet legally established

## Supporting Information

S1 Text INTERVIEW GUIDE. (DOC)

S1 Table CHECKLIST. (DOC)

## References

[1] Stevens P. Diseases of poverty and the 10/90 Gap IPN Working Papers on Intellectual Property, Innovation and Health. 2004; London, UK Retrieved 26 May 2016 from http://www.who.int/entity/intellectualproperty/submissions/en/InternationalPolicyNetwork.pdf

[2] World Health Organisation [interent]. Global report for research on infectious diseases of poverty. 2012; Retrieved 17 September 2015 from http://apps.who.int/iris/bitstream/10665/44850/1/9789241564489_eng.pdf

[3] CCAM. About malaria: For a malaria free Cameroon. A bilingual publication of Cameroon Coalition Against Malaria. 2009;2(1):1–28.

[4] MINSANTE. Profil des Estimations et Projections en Matière de VIH et SIDA au Cameroun 2010–2020. Yaoundé: Ministère de la Santé Publique; 2009 p. 17.

[5] Pefura-Yone EW, Kengne AP, Kuaban C. Non-conversion of sputum culture among patients with smear positive pulmonary tuberculosis in Cameroon: a prospective cohort study. BMC Infect Dis. 2014;14(1):1–6. 24618155 10.1186/1471-2334-14-138 PMC3984706

[6] Ahmed SM, Adams AM, Chowdhury M, & Bhuiya A. Changing health-seeking behaviour in Matlab, Bangladesh: Do development interventions matter? Health Policy Plan. 2003; 18(3): 306–315. 12917272 10.1093/heapol/czg037

[7] Burtscher D & Burza S. Health-seeking behaviour and community perceptions of childhood undernutrition and a community management of acute malnutrition (CMAM) programme in rural Bihar, India: A qualitative study. Public Health Nutr 2015; 10.1017/S1368980015000440 PMC10271539 25753193

[8] Halperin D & Allen A. Is poverty the root cause of African AIDS? AIDS Anal Afr 2001; 11(4): 1–3.

[9] Lozano R, Naghavi M, Foreman K, Lim S, Shibuya K, Aboyans V, Murray CJL. Global and regional mortality from 235 causes of death for 20 age groups in 1990 and 2010: A systematic analysis for the Global Burden of Disease Study 2010. The Lancet, 2012; 380(9859): 2095–2128. 10.1016/S0140-6736(12)61728-0 PMC10790329 23245604

[10] Chen M T, Li C. Y, Lin HC, Shen WW, Hsieh PC, & Chen CC. Health-seeking behavior, alternative medicine, and quality of life in Taiwanese panic disorder patients. Int J Psychiatry Med 2013 17(3): 206–215. 10.3109/13651501.2012.713111 22809126

[11] Chenge MF, Van Der Vennet J, Luboya NO, Vanlerberghe V, Mapatano MA, & Criel B. Health-seeking behaviour in the city of Lubumbashi, Democratic Republic of the Congo: Results from a cross-sectional household survey. BMC Health Serv. Res. 2014; 14(1). 10.1186/1472-6963-14-173 PMC4016631 24735729

[12] Mackian S, Bedri N & Lovel H. Up the garden path and over the edge: Where might health-seeking behaviour take us? Health Policy Plan. 2004; 19(3): 137–146. 15070862 10.1093/heapol/czh017

[13] Antonovsky A. The salutogenic model as a theory to guide health promotion. Health Promot Int, 1996; 11(1): 11–18.

[14] Eriksson M. Unravelling the mystery of salutogenesis: The evidence base of the salutogenic research as measured by Antonovskys Sense of Coherence Scale Doctoral thesis in social policy at ÅAU, 2007 Vasa: Faculty of Social and Caring Science.

[15] Lindström B, & Eriksson M. Contextualizing salutogenesis and Antonovsky in public health development. Health Promot Int. 2006; 21(3): 238–244. 10.1093/heapro/dal016 16717056

[16] Audu O, Bako Ara I, Abdullahi Umar A, Nanben Omole V & Avidime S. Sociodemographic correlates of choice of health care services in six rural communities in North Central Nigeria. Adv Public Health 2014; 7.

[17] Imbahale SS, Fillinger U, Githeko A, Mukabana WR, & Takken W. An exploratory survey of malaria prevalence and people's knowledge, attitudes and practices of mosquito larval source management for malaria control in western Kenya. Acta Tropica 2010; 115(3): 248–256. 10.1016/j.actatropica.2010.04.005 20399739

[18] Musoke D, Karani G, Ssempebwa JC & Musoke MB (2013). Integrated approach to malaria prevention at household level in rural communities in Uganda: Experiences from a pilot project. Malar J. 2013; 12(1). 10.1186/1475-2875-12-327 PMC3848758 24041445

[19] Abosede OA. Self-medication: An important aspect of primary health care. Soc. Sci. Med. 1984 19(7), 699–703. 6505739 10.1016/0277-9536(84)90242-9

[20] Friend-du Preez N, Cameron N & Griffiths P. "So they believe that if the baby is sick you must give drugs." The importance of medicines in health-seeking behaviour for childhood illnesses in urban South Africa. Soc. Sci. Med. 2013; 92: 43–52. 10.1016/j.socscimed.2013.05.014 23849278

[21] Chipwaza B, Mugasa JP, Mayumana I, Amuri M, Makungu C & Gwakisa PS. Self-medication with anti-malarials is a common practice in rural communities of Kilosa district in Tanzania despite the reported decline of malaria. Malar J. 2014; 13(1): 1–11. 24992941 10.1186/1475-2875-13-252 PMC4087197

[22] Kahabuka C, Moland KM, Kivale G & Hinderaker SG. Unfulfilled expectations to services offered at primary health care facilities: Experiences of caretakers of underfive children in rural Tanzania. BMC Health Serv. Res. 2012; 12 158 10.1186/1472-6963-12-158 22697458 PMC3420314

[23] Shaikh BT & Hatcher J. Health seeking behaviour and health service utilization in Pakistan: Challenging the policy makers. J. Public Health. 2005; 27(1): 49–54. 10.1093/pubmed/fdh207 15590705

[24] Steenkamp V. Traditional herbal remedies used by South African women for gynaecological complaints. J Ethnopharmacol. 2003; 86(1): 97–108. 12686447 10.1016/s0378-8741(03)00053-9

[25] Stickel F & Schuppan D. Herbal medicine in the treatment of liver diseases. Dig Liver Dis. 2007; 39(4): 293–304. 10.1016/j.dld.2006.11.004 17331820

[26] Helman CG. Culture, Health and Illness. 2007 (5th ed.). London: Hodder Arnold.

[27] Makoge V, Maat H, Edward N, & Emery J. Knowledge, attitudes and practices towards malaria in Mbonge and Kumba sub-divisions in Cameroon. Int J Trop Dis Health. 2016; 15(2): 1–13.

[28] Sabuni LP. Dilemma with the local perception of causes of illnesses in Central Africa: Muted concept but prevalent in everyday life. Qual. Health Res. 2007; 17(9): 1280–1291. 10.1177/1049732307307864 17968044

[29] Asampong E, Dwuma-Badu K, Stephens J, Srigboh R, Neitzel R, Basu N & Fobil JN. Health seeking behaviours among electronic waste workers in Ghana. BMC Public Health. 2015; 15(1): 1065. 26474859 10.1186/s12889-015-2376-z PMC4609051

[30] Biswas P, Kabir ZN, Nilsson J & Zaman S. Dynamics of health care seeking behaviour of elderly people in rural Bangladesh. International Journal of Ageing and Later Life. 2006; 1(1): 69–89.

[31] Stevenson FA, Britten N, Barry CA, Bradley CP & Barber N. Self-treatment and its discussion in medical consultations: How is medical pluralism managed in practice? Soc. Sci. Med. 2003; 57(3): 513–527. 12791493 10.1016/s0277-9536(02)00377-5

[32] Abdulraheem IS & Parakoyi DB. Factors affecting mothers’ healthcare‐seeking behaviour for childhood illnesses in a rural Nigerian setting. Early Child Dev Care 2009; 179(5): 671–683.

[33] Cameroon Development Corporation [Internet]. CDC. 2009; Retrieved July 2014 from http://www.cdc-cameroon.com/Subpages/

[34] University of Buea. [Internet]. About UB. 2014; Retrieved 15 August 2015 from http://www.ubuea.cm/about/

[35] Institut National de la Statistique [Internet]. Annuaire Statistique du Cameroun. 2010; Retrieved from http://www.statistics-cameroon.org/downloads/annuaire2010/chap6.pdf.

[36] Strauss A, Corbin J. Basics of qualitative research: Techniques and procedures for developing grounded theory. Sage Publications, Inc; Thousand Oaks Califormia,1998.

[37] Antonovsky A. Health, stress, and coping. 1979; San Francisco, CA: Jossey-Bass.

[38] Antonovsky A. Unraveling the mystery of health: How people manage stress and stay well. 1987; San Francisco, CA: Jossey-Bass.

[39] Grundy J, & Annear P. Health-seeking behaviour studies: A literature review of study design and methods with a focus on Cambodia Health Policy and Health Finance Knowledge Hub Working Paper Series No. 7. Melbourne, Australia: The Nossal Institute for Global Health 2010

[40] MacKian S. A review of health seeking behaviour: Problems and prospects. 2003; Manchester, UK: The University of Manchester, Health Systems Development Programme.

[41] Braun V & Clarke V. Using thematic analysis in psychology. Qual Res Psychol. 2006; 3(2): 77–101.

