# Supplementary material for: Health-Seeking Behaviour towards Poverty-Related Disease (PRDs): A Qualitative Study of People Living in Camps and on Campuses in Cameroon
Source: PLoS Negl Trop Dis. 2017 Jan 4;11(1):e0005218. doi: 10.1371/journal.pntd.0005218 (PMC5214973; doi:10.1371/journal.pntd.0005218)
Supplement: S1 Text — (DOC) [file pntd.0005218.s001.doc]

**Interview Guide**

**Introduction:** the interview will start with an introduction (of researcher), then explanation of the purpose of the research and then signing of informed consent. The interview will take about 30-90minutes and will be recorded and later transcribed. The salutogenic model proposes that the goal of health research should be to identify, define and describe pathways, factors and causes of positive health to supplement our knowledge about how to prevent, treat and manage negative health (pathogenesis)

What meaning do people give to resources for health?

**Part One: Life story and Experiences**

Camps

1. What is your current position or occupation? How long have you been doing what you do now? Can you briefly tell me what you do in your work?
2. What motivates you about your work?
3. What led you to do this job? What were you doing before you started working for CDC. What attracted you to work here?
4. Where did you grow up? School? Class? What was it like when you were in school?
5. Key models or people who deeply influenced who you are, what you believe in and what you’re committed to in your work and life?  Tell me about them.
6. Occupational health
   1. Are there any risky activities at work? If so, which ones
   2. What are the protective measures taken at work to ensure that you don’t have an accident
   3. Do you sometimes suffer from cuts and bruises
   4. How do you manage in such situations?

Campuses:

1. How long have you been in the University? In which department are you? Can you tell me briefly about your department?
2. What motivates you about the course you are reading? What are your goals personally?
3. What attracted you to the course you are doing now? what did you do in high school.(this is in case the student just graduated. If working? Question will be asked what motivated them to come back to the university
4. What do you like about the course you are reading in the university?
5. Where did you grow up? What was it like when you were in high school? How is it different from the university or is it the same?
6. Did you have key models or people who deeply influenced who you are, what you believe in and what you are committed to in school and life? Tell me about them?
7. Who is paying for your education? If parents, what is the occupation of the parents?
8. Describe neighbourhood? What do you like about it

**Part two: The poverty related disease story:**

1. What are the illnesses or diseases that are present here?
2. Which ones are more common?
3. Which ones are related to poverty?
4. What happens when someone is sick? When will you go to the hospital? (why?) When will you not go to the hospital? (why?)
5. What is good health?
6. **Self-rated health: How is your health in general? Options are very good, good, fair, poor very poor**
7. Have you sought health care in the last month? which and why?
8. Have you bought medicines or taking herbs for malaria during the last 4 weeks?
9. What would determine that you seek health care? ( Age, gender, distance of health care service, length of illness, and rated severity of illness)
10. How would you define or describe your quality of life and wellbeing?
11. Do you have a health plan? How does living in this neighborhood fit with your health plan?
12. Are your neighbours health conscious?
13. What do they do to maintain their health? What about your classmates? And other people you know? Like family?
14. What does the university provide to ensure good health for the students? How does it work? What’s the student response?
15. In your opinion how do students in Molyko (or Ngoa Ekelle) keep healthy.
16. poverty related diseases?
17. Are there any diseases occurring here that you would describe as poverty related?
18. Which are they and why do you describe them as being poverty- related?
19. What do you do to ensure that you stay in good health? How do you maintain good health?
20. What activities do you take part in in ensuring that you have good health
21. Are there certain foods that you eat for health
22. What other practices do you undertake for health
23. What sanitary or hygienic practices do you do for health
24. Who would you depend on for your health?
25. What services are available for health care? How do they work?
26. Are they affordable? explain
27. Are you confident that your health provider is experienced enough to handle any disease you present?
28. How far is the health service from where you stay?
29. What would you associate with good health(flexibility, stability)

**Part three: Stressors and resources**

1. How would you describe life in this camp or on campus?
2. What aspects or things can you describe as being a cause of stress here ?( education, job , poverty, health etc)
3. What would you do when you are faced with a stressing situation? Who would you count on? (yourself, neighbour, family, church etc)
4. Do you belong to a church, church group, traditional meeting, or any kind of association?
5. What made you join(if previous answer is yes)
6. What are the benefits of belonging to any of the above?
7. What are the determinants of health according to you? What would either give you or keep you from good health?
8. What strong points do you use to become well or keep well?(self-assessed health resources)
9. Do you know any people who are always sick in your community? Why do you think that happens?
10. Do you know people who are hardly ever sick in your community? What do you think they do to stay that way?
11. What are your sources of strength in the face of stressors
12. What do you do for leisure?
13. How can a person move towards great health
