# Supplementary material for: Health-Seeking Behaviour towards Poverty-Related Disease (PRDs): A Qualitative Study of People Living in Camps and on Campuses in Cameroon
Source: PLoS Negl Trop Dis. 2017 Jan 4;11(1):e0005218. doi: 10.1371/journal.pntd.0005218 (PMC5214973; doi:10.1371/journal.pntd.0005218)
Supplement: S1 Table — (DOC) [file pntd.0005218.s002.doc]

STROBE Statement—Checklist of items that should be included in reports of ***cross-sectional studies***

| **Section/Topic** | Item No | Checklist Item | Reported on page number |  |  |
| --- | --- | --- | --- | --- | --- |
| **Title and abstract** | 1 | (*a*) Indicate the study’s design with a commonly used term in the title or the abstract | (a) and (b) done in title and abstract of manuscript |  |  |
|  |  | (*b*) Provide in the abstract an informative and balanced summary of what was done and what was found | Done |  |  |
| Introduction | | |  |  |  |
| Background/rationale | 2 | Explain the scientific background and rationale for the investigation being reported | page 2 |  |  |
| Objectives | 3 | State specific objectives, including any prespecified hypotheses | page 3 |  |  |
| Methods | | |  |  |  |
| Study design | 4 | Present key elements of study design early in the paper | page 5 |  |  |
| Setting | 5 | Describe the setting, locations, and relevant dates, including periods of recruitment, exposure, follow-up, and data collection | page 4 |  |  |
| Participants | 6 | (*a*) Give the eligibility criteria, and the sources and methods of selection of participants | page 5 |  |  |
| Variables | 7 | Clearly define all outcomes, exposures, predictors, potential confounders, and effect modifiers. Give diagnostic criteria, if applicable | n/a |  |  |
| Data sources/ measurement | 8 | For each variable of interest, give sources of data and details of methods of assessment (measurement). Describe comparability of assessment methods if there is more than one group | Page 5 |  |  |
| Bias | 9 | Describe any efforts to address potential sources of bias | page 24 |  |  |
| Study size | 10 | Explain how the study size was arrived at | page 6 |  |  |
| Quantitative variables | 11 | Explain how quantitative variables were handled in the analyses. If applicable, describe which groupings were chosen and why | n/a |  |  |
| Statistical methods | 12 | (*a*) Describe all statistical methods, including those used to control for confounding | n/a |  |  |
|  |  | (*b*) Describe any methods used to examine subgroups and interactions |  |  |  |
|  |  | (*c*) Explain how missing data were addressed |  |  |  |
|  |  | (*d*) If applicable, describe analytical methods taking account of sampling strategy |  |  |  |
|  |  | (*e*) Describe any sensitivity analyses |  |  |  |
| Results | | |  |  |  |
| Participants | 13 | (a) Report numbers of individuals at each stage of study—eg numbers potentially eligible, examined for eligibility, confirmed eligible, included in the study, completing follow-up, and analysed | page 5 |  |  |
|  |  | (b) Give reasons for non-participation at each stage | n/a |  |  |
|  |  | (c) Consider use of a flow diagram | n/a |  |  |
| Descriptive data | 14 | (a) Give characteristics of study participants (eg demographic, clinical, social) and information on exposures and potential confounders | page 5 |  |  |
|  |  | (b) Indicate number of participants with missing data for each variable of interest | n/a |  |  |
| Outcome data | 15 | Report numbers of outcome events or summary measures | n/a |  |  |
| Main results | 16 | (*a*) Give unadjusted estimates and, if applicable, confounder-adjusted estimates and their precision (eg, 95% confidence interval). Make clear which confounders were adjusted for and why they were included | n/a |  |  |
|  |  | (*b*) Report category boundaries when continuous variables were categorized | n/a |  |  |
|  |  | (*c*) If relevant, consider translating estimates of relative risk into absolute risk for a meaningful time period | n/a |  |  |
| Other analyses | 17 | Report other analyses done—eg analyses of subgroups and interactions, and sensitivity analyses | page 7-8 |  |  |
| Discussion | | |  |  |  |
| Key results | 18 | Summarise key results with reference to study objectives | page 19 |  |  |
| Limitations | 19 | Discuss limitations of the study, taking into account sources of potential bias or imprecision. Discuss both direction and magnitude of any potential bias | page 24 |  |  |
| Interpretation | 20 | Give a cautious overall interpretation of results considering objectives, limitations, multiplicity of analyses, results from similar studies, and other relevant evidence | page 19 |  |  |
| Generalisability | 21 | Discuss the generalisability (external validity) of the study results | page 24 |  |  |
| Other information | | |  |  |  |
| Funding | 22 | Give the source of funding and the role of the funders for the present study and, if applicable, for the original study on which the present article is based | This work was funded by Wageningen University. The funders had no role in the study design, data collection and analysis, decision to publish, or preparation of the  manuscript |  |  |
